# *In Silico* Assigned Resistance Genes Confer *Bifidobacterium* with Partial Resistance to Aminoglycosides but Not to Β-Lactams

**DOI:** 10.1371/journal.pone.0082653

**Published:** 2013-12-06

**Authors:** Fiona Fouhy, Mary O’Connell Motherway, Gerald F. Fitzgerald, R. Paul Ross, Catherine Stanton, Douwe van Sinderen, Paul D. Cotter

**Affiliations:** 1 Teagasc Food Research Centre, Moorepark, Fermoy, Cork, Ireland; 2 Microbiology Department, University College Cork, Cork, Ireland; 3 Alimentary Pharmabiotic Centre, Cork, Ireland; Ghent University, Belgium

## Abstract

Bifidobacteria have received significant attention due to their contribution to human gut health and the use of specific strains as probiotics. It is thus not surprising that there has also been significant interest with respect to their antibiotic resistance profile. Numerous culture-based studies have demonstrated that bifidobacteria are resistant to the majority of aminoglycosides, but are sensitive to β-lactams. However, limited research exists with respect to the genetic basis for the resistance of bifidobacteria to aminoglycosides. Here we performed an in-depth *in silico* analysis of putative *Bifidobacterium*-encoded aminoglycoside resistance proteins and β-lactamases and assess the contribution of these proteins to antibiotic resistance. The *in silico*-based screen detected putative aminoglycoside and β-lactam resistance proteins across the *Bifidobacterium* genus. Laboratory-based investigations of a number of representative bifidobacteria strains confirmed that despite containing putative β-lactamases, these strains were sensitive to β-lactams. In contrast, all strains were resistant to the aminoglycosides tested. To assess the contribution of genes encoding putative aminoglycoside resistance proteins in *Bifidobacterium* sp. two genes, namely *Bbr_0651* and *Bbr_1586*, were targeted for insertional inactivation in *B. breve* UCC2003. As compared to the wild-type, the UCC2003 insertion mutant strains exhibited decreased resistance to gentamycin, kanamycin and streptomycin. This study highlights the associated risks of relying on the *in silico* assignment of gene function. Although several putative β-lactam resistance proteins are located in bifidobacteria, their presence does not coincide with resistance to these antibiotics. In contrast however, this approach has resulted in the identification of two loci that contribute to the aminoglycoside resistance of *B. breve* UCC2003 and, potentially, many other bifidobacteria.

## Introduction

Following the discovery of penicillin by Alexander Fleming [1], exponential antibiotic discovery and development occurred which revolutionized medicine. However, during this same period, target bacteria developed sophisticated mechanisms of resistance against many of the most commonly prescribed antibiotics [2]. It is thus not surprising that considerable efforts have been and are still being made to investigate the genetic mechanisms involved in the transfer, acquisition and expression of antibiotic resistance genes, in order to curtail or prevent the further development of resistance [3,4].

The mechanisms underlying resistance to aminoglycosides and to β-lactams are among those that have been the focus of particular attention. Briefly, aminoglycosides are a family of broad spectrum antibiotics that were first reported in 1944 [5], whose bactericidal activity results from their binding to the 30S subunit of the prokaryotic ribosome and the subsequent impairment of protein synthesis [5,6]. Aminoglycoside resistance can be mediated through reduced aminoglycoside uptake [7], or through enzymatic modification of the aminoglycoside through the activity of the *N*-acetyltransferases (AAC), *O*-nucleotidyltransferases (ANT) or *O*-phosphotransferases (APH). Aminoglycoside resistance genes have been classified based on the enzymatic modification mechanism used by the resultant protein and the chemical position at which the aminoglycoside is modified [8].

β-lactam antibiotics are a class of broad spectrum antibiotics which include the penicillins and cephalosporins [9]. β-lactams inhibit bacteria by their interference with normal cell wall synthesis, via disruption of the final cross-linking stage of cell wall peptidoglycan formation, resulting in a significantly weakened cell wall polymer, ultimately leading to bacterial cell death [10-12]. β-lactam resistance can arise through mutation of target penicillin binding proteins (PBPs; [13,14]), as well as through the production of β-lactamases [15], which catalyze the hydrolysis of the eponymous β-lactam rings present in β-lactam antibiotics, rendering the antibiotic inactive. β-lactamase classification has undergone significant rounds of change from the initial Ambler classification proposed in 1973 [16] and the classification schemes of Bush and colleagues [17-20].

The antibiotic resistance genes of pathogenic bacteria have been the focus of greatest attention. Similarly, antibiotic sensitivity is regarded as a desirable trait among candidate probiotic strains for the feed [21] and human [22,23] markets. Such a phenotype ensures that their consumption does not further increase the risk of antibiotic resistance gene dissemination, especially in situations where such genes are located on mobile genetic elements. Gut-associated bifidobacteria are generally viewed as beneficial microbes and many strains have been attributed with health-promoting characteristics [24-27]. Thus, it is not surprising that many bifidobacteria are used, or have been studied with a view to their potential use, as probiotics in functional foods [28]. As a consequence, there has been considerable interest in determining if certain bifidobacteria possess antibiotic resistance genes [29-32]. These studies established that the tested bifidobacteria strains are generally resistant to aminoglycoside antibiotics [33], but are sensitive to β-lactams [29,31,34,35]. In a previous study, we found that combined ampicillin and gentamycin treatment in infants, caused a significant decrease in the proportion of bifidobacteria present 4 weeks after antibiotic administration ceased, while also significantly altering the bifidobacteria species present [36]. We were therefore interested in investigating differences in the distribution of genes encoding β-lactam or aminoglycoside resistance proteins among members of the *Bifidobacterium* genus.

To date little is known about the genetic mechanisms that underlie aminoglycoside resistance in bifidobacteria. Despite the existence of some specific studies [32,37,38], the presence of antibiotic resistance genes has been more frequently inferred through the annotation of DNA sequences and the identification of genes bearing some homology to genes previously assigned as being potential resistance determinants. Given the risks associated with relying exclusively on rapid *in silico* assignments, here we present an in-depth bioinformatic analysis of putative β-lactam and aminoglycoside resistance proteins that are *Bifidobacterium*-encoded. We have investigated if a correlation exists between these proteins and antibiotic resistance and, in the case of aminoglycoside resistance, have demonstrated the contribution of the assigned resistance genes to this phenotype. 

## Materials and Methods

### NCBI database search for *Bifidobacterium*-associated β-lactam and aminoglycoside resistance proteins

Using the NCBI protein database, a search for putative β-lactamases and aminoglycoside resistance proteins associated with bifidobacteria was completed using the terms ‘beta-lactamase’ and ‘*Bifidobacterium*’ (searched on 28/8/12) and ‘aminoglycoside’ and ‘*Bifidobacterium*’ (search completed on 29/8/12). This approach was taken so that all such proteins, regardless of the basis upon which they were assigned, would be revealed. Following the removal of duplicates and sequences that did not originate from *Bifidobacterium*, all remaining sequences were used as drivers for subsequent rounds of BLAST investigations. All subsequent distinct sequences detected were employed for additional BLAST-based investigations until a finalized list was achieved. Additionally, further BLAST-based investigations using known β-lactamase and aminoglycoside resistance proteins as drivers were completed to ensure no additional sequences were overlooked. 

### Classification of β-lactamases and aminoglycoside resistance protein sequences from bifidobacteria

Putative *Bifidobacterium*-associated β-lactamase and aminoglycoside resistance proteins were subjected to *in silico* analysis with a view to classifying them using the Ambler method for β-lactamases [17], or assigning them into one of the 3 main enzyme modification groups associated with aminoglycoside resistance [8]. To this end, the putative *Bifidobacterium*-associated resistance determinants were aligned (MegAlign Clustal W, LaserGene) against representative sequences from each class (A-D for the β-lactamases) and from each of the 3 enzyme groups (AAC, APH and ANT for the aminoglycosides) [19,20] (Table 1). 

**Table 1:** Representative sequences used as drivers for Blast based investigations into *Bifidobacterium*-associated aminoglycoside resistant proteins and β-lactamases.

**Aminoglycoside resistance gene classification groups**	**Representative sequences**	**β-lactamase gene classes**	**Representative gene name**	**Representative gene accession number**
**APH**	M20305	**Class A**	TEM1	YP_209323.1
	V00618		TEM1	AFN82055.1
	M29953		SHV-2	YP_001966240.1
	X07753		PSE	YP_005086938.1
**APH (6’)**	X05648		CepA	YP_210868.1
	X01702		Sme_1	CAA82281.1
**AAC 3**	X01385		Bla KPC	YP_003754012.1
	M55426	**Class B**	IMP-1	YP_005980003.1
	M22999		VIM-1	YP_003813035.1
**AAC-Ia & Ib**	L06157		CcrA	YP_004735262.1
**AAC 6’ Ic**	M94066		L1	YP_006185056.1
**ANT**	X02340		CphA	YP_004391384.1
	X04555		Sph1	YP_005188946.1
		**Class C**	AMP C	AAG59351.1
		**Class D**	OXA-1	AFB82783.1
			OXA-10	YP_001715358.1
			OXA-23	YP_002317955.1

### Laboratory based assessments of antibiotic resistance

The antibiotic susceptibility of bifidobacteria strains was investigated in a number of different ways. Disc diffusion assays were carried out according to the British Society for Antimicrobial Chemotherapy (BSAC) guidelines [39-41]. Briefly the bifidobacteria strains were cultured overnight anaerobically and delivered onto Iso-Sensitest agar plates (Oxoid, Fisher Scientific, Dublin, Ireland) using a swab in three directions. Antimicrobial discs containing ampicillin (25 µg), penicillin (10 µg) (VWR International, Dublin, Ireland), neomycin (30 µg), gentamycin (200 µg), kanamycin (30 µg) and streptomycin (25 µg) (Fisher Scientific, Dublin, Ireland) were dispensed manually onto the agar plates. Following anaerobic incubation at 37°C for 48 hours, the diameters of the zones of inhibition (mm) were measured. All tests were carried out in triplicate.

Minimum inhibitory concentration tests (MICs) using 4 aminoglycosides i.e. neomycin, gentamycin, streptomycin and kanamycin (Sigma Aldrich, Dublin, Ireland) were performed as per the micro-dilution method, as described in detail by others [42]. Briefly, bifidobacteria were grown overnight anaerobically at 37°C in MRS broth supplemented with 0.05% cysteine (Sigma Aldrich, Wexford, Ireland). Cultures were adjusted to an OD_600_ of 0.1 (≈ 1 x 10^5^ cfu/ml) in fresh MRS broth (media pH 6.8). Stock solutions of each of the aminoglycoside antibiotics were prepared in sterile distilled water and a 2-fold dilution series was performed. An inoculum of 100 µl of culture was added to each well of the 96 well plate (resulting in a final concentration of ≈ 5 x 10^4^ cfu/ml) (Sarstedt, Wexford, Ireland). Additionally, each 96 well plate contained positive (MRS + culture) and negative controls (MRS only), and tests were carried out in triplicate. Plates were incubated anaerobically (using anaerobic gas jars and Anaerocult P anaerobic gas pack inserts (Merck Millipore Ltd, Cork, Ireland)) at 37°C for 24 hours and the MIC was determined as the lowest concentration of antimicrobial agent at which no visible growth was recorded. MICs were also carried out on *E. coli* XL1-blue which had been transformed with plasmid-encoded copies of the putative aminoglycoside resistance genes *Bbr_0651*, *Bbr_1586* and *Bbr_0651+0650*. Protocols were as described above except that LB broth (pH 7.1) (Difco, Fisher Scientific, Ireland) was used for culturing and growth conditions were 24 hours aerobically at 37°C.

To test for β-lactamase activity, nitrocefin tests were performed as previously described [43,44], i.e. β-lactamase nitrocefin sticks (Fisher Scientific, Ireland), were dipped into a single colony for each species being tested and assessed for 1-2 minutes and again after 15 minutes for the appearance of a pink colour, indicative of β-lactamase activity. *Staphylococcus aureus* DPC 5286 was used as the positive control. 

### Disruption of the *Bbr_0651* and *Bbr_1586* genes from *B. breve* UCC2003

Site specific homologous recombination was used to disrupt 2 genes present in *B. breve* UCC2003, namely *Bbr_0651* and *Bbr_1586*, using protocols similar to those previously described [45,46]. Briefly, internal fragments of *Bbr_0651* and *Bbr_1586*, were amplified by PCR using specifically designed primers (MWG Eurofins, Germany) (Table S1), resulting in 500bp and 400bp products respectively. These fragments were cloned into the pORI19 vector and a tetracycline resistance marker (*tetW* gene) from the pAM5 vector [47] was subcloned to generate the plasmids pORI19-tet-0651 and pORI19-tet-1586 (Table 2). The correct sequence of each cloned insert was verified by sequencing (Source BioScience, Dublin, Ireland).

**Table 2:** Bacterial strains and plasmids used in this study.

**Strain or plasmid**	**Relevant characteristics**	**Ref or Source**
***E.coli* strains**		
EC101	Cloning host, repA**^*+*^** , kan**^*r*^**	Law et al. (1995)
XL1-blue	Tet**^*r*^**	Stratagene
XL1-blue-pBC1.2-Bbr_0651	Heterologous expression of *Bbr_0651*	This study
XL1-blue-pBC1.2-Bbr_0651+0650	Heterologous expression of *Bbr_0651+0650*	This study
XL1-blue-pBC1.2-Bbr_1586	Heterologous expression of *Bbr_1586*	This study
***B. breve* strains**		
UCC2003	Isolated from nursing stool	Mazé et al. (2007)
UCC2003-0651-tet	pORI19-0651-tet insertion mutant of *B. breve* UCC2003	This study
UCC2003-1586-tet	pORI19-1586-tet insertion mutant of *B. breve* UCC2003	This study
*B. breve* UCC2003-gosG	pORI19-tet-Bbr_0529 insertion mutant of UCC2003	O’ Connell Motherway et al. (2013)
UCC2003-1586-tet-pBC1.2-Bbr_1586	pORI19-1586-tet insertion mutant complemented strain of *B. breve* UCC2003	This study
UCC2003-pBC1.2-Bbr_0651	pBC1.2-Bbr_0651 construct in *B. breve* UCC2003	This study
UCC2003-pBC1.2-Bbr_0651+0650	pBC1.2-Bbr_0651+0650 construct in *B. breve* UCC2003	This study
UCC2003-pBC1.2-Bbr_1586	pBC1.2-Bbr_1586 construct in *B. breve* UCC2003	This study
UCC2003-pBC1.2	*B. breve* UCC2003 harbouring pBC1.2	This study
**Bifidobacteria strains**		
*B. gallicum* DSM 20093	Contains putative β-lactamase protein	Teagasc Culture Collection
*B. animalis* subsp. *lactis Bb12*	Contains putative β-lactamase and AG resistance proteins	Teagasc Culture Collection
*B. angulatum DSM 20098*	Contains putative β-lactamase and AG resistance proteins	Teagasc Culture Collection
*B*. *pseudocatenulatum* DSM 20438	Contains putative β-lactamase and AG resistance proteins	Teagasc Culture Collection
*B. breve* DSM 20213	Contains putative β-lactamase and AG resistance proteins	Teagasc Culture Collection
*B. breve* UCC2003	Contains putative β-lactamase and AG resistance proteins	Teagasc Culture Collection
**Plasmids**		
pAM5	pBC1-puC19-Tc**^*r*^**	Alvarez-Martín et al. (2007)
pORI19	Em^r^, repA^-^, ori**^*+*^**, cloning vector	Law et al. (1995)
pORI19-tet-0651	Internal 500bp fragments of *Bbr_0651* and tetW cloned in pORI19	This study
pORI19-tet-1586	Internal 400bp fragments of *Bbr_1586* and tetW cloned in pORI19	This study
pBC1.2	pBC1-pSC101-Cm^r^	Alvarez-Martín et al. (2007)
pBC1.2-0651	*Bbr_0651* cloned in pBC1.2	This study
pBC1.2-0651+0650	*Bbr*_*0651*+*Bbr*_*0650* cloned in pBC1.2	This study
pBC1.2-1586	*Bbr_1586* cloned in pBC1.2	This study

AG: aminoglycoside

Being derivatives of pORI19 these plasmids cannot replicate in *B. breve* UCC2003, due to a lack of a functional replication protein [48], and instead are utilised with a view to integrating into and disrupting target genes. To facilitate methylation, the pORI19 plasmids were introduced via electroporation into EC101 *E. coli* cells containing pNZ-M.BbrII-M.BbrIII. The resulting methylated pORI19-tet-0651 and pORI19-tet-1586 constructs were electroporated into *B. breve* UCC2003. Transformants were selected based on presence of tetracycline resistance. Transformants were expected to carry *Bbr_0651* or *Bbr_1586* gene disruptions, respectively. To verify the suspected chromosomal integration of these pORI19 constructs, colony PCRs were performed on a selection of tetracycline resistant transformants, using a forward primer upstream of the integration region and a reverse primer based on pORI19 (Table S1). 

### Complementation studies

DNA fragments containing the gene *Bbr_1586* and its native promoter region were generated by PCR amplification from *B. breve* UCC2003 chromosomal DNA, using Pfu Ultra II Hotstart Mastermix (Agilent Technologies, Cork, Ireland) and sequence specific primers (Table S1). The amplicons and the pBC1.2 plasmid were digested with *Hin*dIII and *Xba*I (Roche Diagnostics, Sussex, UK) and subsequently ligated using T4 DNA ligase (Roche Diagnostics, Sussex, UK). This resulted in the complementation plasmid pBC1.2-Bbr_1586 (Table 2). The dialysed ligations were electroporated into *E. coli* XL1-blue and the resulting plasmids verified by PCR and restriction digest analysis. Finally, the plasmid pBC1.2-Bbr_1586 was electroporated into competent *B. breve* UCC2003-1586-tet cells. Transformants from the complemented strain were selected and the presence of the construct confirmed.

### Studies of wild-type *B. breve* UCC2003 with additional copies of aminoglycoside resistance genes

Studies were also completed to investigate if the addition of extra plasmid-encoded copies of the putative aminoglycoside resistance genes *Bbr_0651*, *Bbr_0651+0650* or *Bbr_1586* would result in enhanced resistance of the wild-type *B. breve* UCC2003. Competent *B. breve* UCC2003 cells were prepared and transformed with the constructs pBC1.2-0651, pBC1.2-0651+0650 or pBC1.2-1586. Transformants were selected and the presence of the plasmid inserts was confirmed.

### Heterologous expression of putative aminoglycoside resistance genes in *E. coli*

Plasmid-encoded copies of the entire putative aminoglycoside resistance genes *Bbr_0651*, *Bbr_0651+0650* and *Bbr_1586*, along with their native promoters were transformed via electroporation into competent *E. coli* XL1-blue. Following confirmation of the presence of the correct plasmid insert in the transformants, MIC assays were completed, using the protocol outlined above.

## Results

### Putative β-lactamases associated with *Bifidobacterium* species

In order to identify *Bifidobacterium*-associated proteins which have been annotated, or possibly mis-annotated, as β-lactamases, the NCBI protein database was screened for *Bifidobacterium*-associated proteins which had been annotated as β-lactamases or which had been noted to contain β-lactamase associated motifs (searched on 28/8/12). The proteins identified were in turn employed as drivers for BLAST analysis (of non-redundant proteins), to identify and assess the distribution of related *Bifidobacterium*-associated proteins. Subsequent rounds of BLAST analysis, employing the related, yet distinct, protein sequences as drivers, ultimately resulted in saturation. To ensure that other potential β-lactamases were not overlooked, further BLAST-based investigations, using known β-lactamase proteins as drivers, were also carried out to screen all publically available *Bifidobacterium* genomes.

The resultant proteins fell into a number of different categories (Table 3). The most common protein was that annotated variably as a metallo-beta-lactamase family protein, a metal-dependent hydrolase or ribonuclease J such as HMPREF0168_0178 from *B. dentium* ATCC 27679. This protein is conserved, at high (>90%) percentage identity, across almost all publically available *Bifidobacterium* genomes and is a member of the protein family 07521 (Pfam07521; RNA-metabolising metallo-beta-lactamases). A considerable number of other proteins are linked by virtue of containing domains typical of Pfam13354 (a β-lactamase enzyme family of proteins). These proteins are not highly conserved, with distinct subgroups such as those represented by HMPREF0168_1872 from *B. dentium* ATCC 27679, BBB_1387 from *B. bifidum* BGN4, BBB_1559 from *B. bifidum* BGN4 and Bbr_0236 from *B. breve* UCC2003, respectively, being apparent. Other unique members of Pfam13354 are BIFADO_ 0224 (*B. adolescentis* L2-32), BLJ0695 (*B. longum* subsp. *longum* JDM 301) and BAD_1308 (*B. adolescentis* ATCC 15703). *B. dentium* genomes also share a conserved protein, representative of Pfam00144 (a β-lactamase family), such as HMPREF0168_1378 from *B. dentium* ATCC 27679. *B. catenulatum* DSM 16992 (BIFCAT_01331) and *B. pseudocatenulatum* DSM 20438 (BIFPSEUDO_02501) also contained proteins from this family (PF00144) which were highly conserved (>90% identity). However, these were distinct from other PF00144 family proteins associated with *B. dentium* ATCC 27679. The remaining protein of potential relevance is Blon_2358 from *B. longum* subsp. *infantis* ATCC 15697. This protein has been assigned as a β-lactamase but, unlike the other proteins referred to above, its closest homologues are not other *Bifidobacterium*-associated proteins but, rather, are proteins that have been found in the genomes of various clostridia, enterococci and lactobacilli. In addition to containing domains corresponding to Pfam07251, this protein is also representative of Pfam12706, i.e. the lactamase_B_2 family of proteins.

**Table 3:** *Bifidobacterium* derived β-lactamase protein sequences.

***Bifidobacterium* strain**	Accession number*	**Gene name**	**Assigned as**	**Pfam**
*B. dentium* ATCC 27679	ZP_07457312.1^a^	HMPREF0168_1872	Conserved hypothetical protein	PF13354
	ZP_07456818.1^b^	HMPREF0168_1378	β-lactamase	PF00144
	ZP_07455619.1^d^	HMPREF0168_0178	Hypothetical protein	Metal dependent hydrolase with PF07521
*B. dentium* Bd1	YP_003359579.1^a^	BDP_0063	Hypothetical protein	PF13354
	YP_003360049.1^b^	BDP_0556	Hypothetical protein	PF00144
	YP_003361167.1^d^	BDP_1754	Hypothetical protein	Metal dependent hydrolase with PF07521
*B. dentium* ATCC 27678	ZP_02917480.1^a^	BIFDEN_00760	Hypothetical protein	PF11354
	ZP_02916953.1^b^	BIFDEN_00213	Hypothetical protein	PF00144
	ZP_02918099.1^d^	BIFDEN_01398	Hypothetical protein	Metal dependent hydrolase with PF07521
*B. gallicum* DSM 20093	ZP_05965566.1^d^	BIFGAL_03078	Metallo-beta-lactamase family protein	Metal dependent hydrolase with PF07521
*B. adolescentis* L2-32	ZP_02027818.1	BIFADO_0224	Hypothetical protein	PF13354
	ZP_02029327.1^d^	BIFADO_01784	Hypothetical protein	PF07521
*B. animalis* subsp. *lactis* Bb12	YP_005575727.1^d^	BIF_01983	Hydrolase	Metal dependent hydrolase with PF07521
*B. animalis* subsp. *animalis* ATCC 25527	YP_006280466.1^d^	BANAN_06475	Hypothetical protein	Metal dependent hydrolase with PF07521
*B. animalis* subsp. *lactis* AD011	YP_002469408.1^d^	BLA_0533	β-lactamase-like protein	Metal dependent hydrolase with PF07521
*B. bifidum* BGN4	YP_006394858.1^f^	BBB_1387	Penicillin binding protein	PF13354
	YP_006395029.1^g^	BBB_1559	β-lactamase	PF13354
	YP_006393888.1^d^	BBB_0414	Ribonuclease J	Metal dependent hydrolase with PF07521
*B. bifidum* NCIMB 41171	ZP_07803038.1^g^	BBNG_01520	Conserved hypothetical protein	PF13354
	ZP_07803204.1^f^	BBNG_01686	β-lactamase	PF13354
	ZP_07801866.1^d^	BBNG_00347	Conserved hypothetical protein	Metal dependent hydrolase with PF07521
*B. bifidum* PRL 2010	YP_003971645.1^g^	BBPR_1582	β-lactamase	PF13354
	YP_003971485.1^f^	BBPR_1404	β-lactamase	PF13354
	YP_003970583.1^d^	BBPR_0437	Metal-dependent hydrolase	Metal dependent hydrolase with PF07521
*B. longum* subsp. *longum* JDM 301	YP_003660997.1	BLJ_0695	β-lactamase	PF13354
*B. adolescentis* ATCC 15703	YP_910171.1	BAD_1308	β-lactamase	PF13354
	YP_910159.1^d^	BAD_1296	Hypothetical protein	PF07521
*B. breve* UCC2003	ABE94945.1^e^	Bbr_0236	Conserved hypothetical protein with β-lactamase motif	PF13354
	ABE95207.1^d^	Bbr_0510	Metal-dependent hydrolase	Metal dependent hydrolase with PF07521
*B. breve* ACS 071 VSch8b	YP_005582166.1^e^	HMPREF9228_0250	Hypothetical protein	PF13354
	YP_005583195.1^d^	HMPREF9228_1387	Hypothetical protein	Metal dependent hydrolase with PF07521
*B. breve* DSM 20213	ZP_06595304.1^e^	BIFBRE_03112	Putative β-lactamase	PF13354
	ZP_06595596.1^d^	BIFBRE_03411	Metallo-beta-lactamase family protein	Metal dependent hydrolase with PF07521
*B. breve* CECT 7263	EHS86772.1^e^	CECT7263_10968	Putative β-lactamase	PF13354
	EHS85412.1^d^	CECT7263_11981	Metallo-beta-lactamase family protein	Metal dependent hydrolase with PF07521
*B. catenulatum* DSM 16992	ZP_03324536.1^c^	BIFCAT_01331	Hypothetical protein	PF00144
	ZP_03324350.1^d^	BIFCAT_01138	Hypothetical protein	Metal dependent hydrolase with PF07521
*B. bifidum* S17	YP_003939138.1^f^	BBIF_1359	β-lactamase	PF13354
	YP_003938240.1^d^	BBIF_0461	Metallo-beta-lactamase domain-containing protein	Metal dependent hydrolase with PF07521
	YP_003939303.1^g^	BBFI_1524	β-lactamase	PF13354
*B. pseudocatenulatum* DSM 20438	ZP_03741949.1^c^	BIFPSEUDO_02501	Hypothetical protein	PF00144
	ZP_03742801.1^d^	BIFPSEUDO_03375	Hypothetical protein	Metal dependent hydrolase with PF07521
*B. longum* NCC2705	NP696361.1^d^	BL_1192	Hypothetical protein	Metal dependent hydrolase with PF07521
*B. longum* subsp. *infantis* ATCC 55813	ZP_03976420.1^d^	HMPREF0175_0795	Metal dependent hydrolase	Metal dependent hydrolase with PF07521
*B. longum* BBMN68	YP_004000557.1^d^	BBMN68_955	Hydrolase	Metal dependent hydrolase with PF07521
*B. longum* DJ010A	YP_001954894.1^d^	BLD_0950	Metallo-beta-lactamase superfamily hydrolase	Metal dependent hydrolase with PF07521
*B. longum* subsp. *longum* JCM 1217	YP_004220181.1^d^	BLLJ_0420	Hypothetical protein	Metal dependent hydrolase with PF07521
*B. longum* subsp. *longum* JDM301	YP_00366798.1^d^	BLJ_0491	β-lactamase domain-containing protein	Metal dependent hydrolase with PF07521
*B. longum* subsp. *infantis* ATCC 15697	YP_005585858.1^d^	BLIJ_2111	Hypothetical protein	Metal dependent hydrolase with PF07521
	YP_002323794.1	BLon_2358	β-lactamase	PF12706 and 07521
*B. animalis* subsp. *lactis* HN019	ZP_02963481.1^d^	BIFLAC_07662	Hypothetical protein	Metal dependent hydrolase with PF07521
*B. gallicum* DSM 20093	ZP_05965566.1^d^	BIFGAL_03078	Metallo-beta-lactamase family protein	Metal dependent hydrolase with PF07521
*B. angulatum* DSM 20098	ZP_04447555.1^d^	BIFANG_02533	Hypothetical protein	Metal dependent hydrolase with PF07521

^*^ Same superscript indicates proteins share >90% sequence percentage identity

### Putative aminoglycoside resistance proteins associated with *Bifidobacterium* species

An identical approach to that taken for the β-lactamases, was taken to identify *Bifidobacterium*-associated proteins which had been annotated, or potentially mis-annotated, as aminoglycoside resistance proteins. A search of the NCBI protein database using the terms ‘aminoglycoside’ and ‘*Bifidobacterium*’ was completed (search completed on 29/8/12). The analysis revealed that putative aminoglycoside resistance proteins are widely distributed across the *Bifidobacterium* genus, and are particularly common among strains of *B. longum* (Table 4). Furthermore, it appears that all putative *Bifidobacterium*-associated aminoglycoside resistance proteins can be broadly classified into 3 groups i.e. those containing proteins of the family Pfam01636 (phosphotransferase enzyme family), proteins containing a protein kinase family domain, c109925, or those which appear to contain both. While some of these proteins appeared to be highly conserved within or across bifidobacteria strains and species, some proteins appear to be much more distantly related. The results indicated that only one putative protein was solely associated with the protein family Pfam01636, namely BBMN_137 from *B. longum* BBMN68. In a number of other instances proteins which were members of Pfam01636 and which also contained the c109925 domain, were noted. In some cases these proteins were annotated as aminoglycoside phosphotransferases, e.g. BIF_01665 (*B. animalis* subsp. *lactis* Bb12), while in other cases they were annotated as desulfatases, e.g. BL_1642 (*B. longum* NCC 2705), or homoserine kinases, e.g. BBMN_1674 (*B. longum* BBMN8). In addition, *B. bifidum* BGN4 BBB_0978 and *B. bifidum* S17 BBIF_0997 also exhibit characteristics of Pfam01636 and possess a protein kinase domain, but have been annotated as an N-acetyl hexosamine kinase and a mucin desulfatase, respectively. In this instance, laboratory-based investigations have previously established that this gene does indeed encode N-acetyl hexosamine kinase [49]. Some sequences which were annotated as being from Pfam01636 and also contained a protein kinase family domain were highly conserved (with >90% percentage identity) e.g. BLD_1766 (*B. longum* DJ010A) and BLIG_01601 from *B. longum* subsp. *infantis* CCUG 52486). However, in other instances, these proteins were more distantly related e.g. BBIF_0997 (*B. bifidum* S17) and Bbr_1586 (*B. breve* UCC2003). 

**Table 4:** *Bifidobacterium* derived aminoglycoside resistance proteins.

***Bifidobacterium* strain**	Accession number*	**Gene name**	**Assigned as**	**Pfam**
*B. longum* DJ010A	YP_00195405.3^a^	BLD_0109	AG phosphotransferase	Proteins containing a protein kinase family domain, c109925
	ZP_00121257.2^a^	Blon_03001154	Hypothetical protein	Proteins containing a protein kinase family domain, c109925
	ZP_00121797.2^b^	BLD_1766	Hypothetical protein	Phosphotransferase family with PF 01636 and proteins containing a protein kinase family domain, c109925
*B. longum* BBMN68	YP_003999751.1^a^	BBMN68_137	AG phosphotransferases	Phosphotransferase family with PF 01636
	YP_004001272.1^b^	BBMN_1674	Homoserine kinase	Proteins containing a protein kinase family domain, c109925 and phosphotransferase family with PF 01636
*B. longum* subsp. *infantis* CCUG 52486	ZP_04663835.1^a^	BLIG_01916	Hypothetical protein	Proteins containing a protein kinase family domain, c109925
	ZP_04664566.1^b^	BLIG_01601	Hypothetical protein	Proteins containing a protein kinase family domain, c109925 and phosphotransferase family with PF 01636
*B. longum* NCC 2705	NP695320.1^a^	BL_0091	Hypothetical protein	Proteins containing a protein kinase family domain, c109925
	NP696793.1^b^	BL_1642	Desulfatase	Proteins containing a protein kinase family domain, c109925 and phosphotransferase family with PF 01636
*B. longum* KACC 91563	YP_005586893.1^a^	BLNIAS_ 00852	Hypothetical protein	Proteins containing a protein kinase family domain, c109925
*B. adolescentis* L2-32	ZP_02029839.1^i^	BIFADO_02300	Hypothetical protein	Proteins containing a protein kinase family domain, c109925
*B. longum* subsp. *infantis* ATCC 55813	ZP_03976875.1^a^	HMPREF0175_1250	AG phosphotransferase	Proteins containing a protein kinase family domain, c109925
*B. longum* subsp. *infantis* ATCC 15697	YP_002322254.1^a^	Blon_0773	AG phosphotransferase	Proteins containing a protein kinase family domain, c109925
	YP_002323612.1^a^	Blon_2173	AG phosphotransferase	Proteins containing a protein kinase family domain, c109925 and phosphotransferase family with PF 01636
*B. longum* subsp. *longum* JDM301	YP_003661654.1^a^	BLJ_1379	AG phosphotransferases	Proteins containing a protein kinase family domain, c109925
*B. breve* UCC2003	ABE95342.1^c^	Bbr_0651	Conserved Hypothetical secreted protein	Merozoite surface protein 1 (MSP1) C-terminus of the PF 07462 and proteins containing a protein kinase family domain, c109925
	ABE96255.1 ^d^	Bbr_1586	AG phosphotransferases	Proteins containing a protein kinase family domain, c109925 and phosphotransferase family with PF 01636
*B. breve* DSM 20213	ZP_06595772.1 ^c^	BIFBRE_03589	Conserved hypothetical protein	Merozoite surface protein 1 (MSP1) C-terminus of the PF 07462 and proteins containing a protein kinase family domain, c109925	
	ZP_06596651.1 ^d^	BIFBRE_04498	Mucin desulfating sulfatase	Proteins containing a protein kinase family domain, c109925 and phosphotransferase family with PF 01636	
*B. breve* CECT 7263	EHS85254.1 ^d^	CECT7263_14691	Mucin desulfating sulfatase	Proteins containing a protein kinase family domain, c109925 and phosphotransferase family with PF 01636	
	EHS85519.1^c^	CECT7263_10981	Hypothetical protein	Merozoite surface protein 1 (MSP1) C-terminus of the PF 07462 and proteins containing a protein kinase family domain, c109925	
*B. breve* ACS 071 VSch 8b	YP_005583039.1^c^	HMPREF9228_1217	Phosphotransferase enzyme domain protein	Merozoite surface protein 1 (MSP1) C-terminus of the PF 07462 and proteins containing a protein kinase family domain, c109925	
	YP_005583418.1^d^	HMPREF9228_1637	Putative mucin-desulfating sulfatase	Proteins containing a protein kinase family domain, c109925 and phosphotransferase family with PF 01636	
*B. animalis* subsp. *lactis* Bb12	YP_005575653.1^e^	BIF_00526	Hypothetical protein	Proteins containing a protein kinase family domain, c109925 and phosphotransferase family with PF 01636	
	YP_005576071.1^f^	BIF_01665	AG 3' phosphotransferase	Proteins containing a protein kinase family domain, c109925 and phosphotransferase family with PF 01636	
*B. dentium* ATCC 27678	ZP_02918244.1^g^	BIFDEN_01548	Hypothetical protein	Proteins containing a protein kinase family domain, c109925 and phosphotransferase family with PF 01636	
*B. dentium* Bd1	YP_003361041.1^g^	BDP_1625	AG phosphotransferase	Proteins containing a protein kinase family domain, c109925 and phosphotransferase family with PF 01636	
*B. dentium* ATCC 27679	ZP_07455726.1^g^	HMPREF0168_0285	Conserved hypothetical protein	Proteins containing a protein kinase family domain, c109925 and phosphotransferase enzyme family of the PF 01636	
*B. dentium* JCVHM P022	ZP_07696282.1^g^	HMPREF9003_0562	Conserved hypothetical protein	Proteins containing a protein kinase family domain, c109925 and phosphotransferase enzyme family of the PF 01636	
*B. catenulatum* DSM 16992	ZP_03323625.1^h^	BIFCAT_00394	Hypothetical protein	Proteins containing a protein kinase family domain, c109925 and phosphotransferase family with PF 01636	
*B. pseudocatenulatum* DSM 20435	ZP_03742521.1^h^	BIFPSEUDO_03094	Hypothetical protein	Proteins containing a protein kinase family domain, c109925 and phosphotransferase family with PF 01636	
*B. adolescentis* ATCC 15703	YP_910027.1^i^	BAD_1164	Hypothetical protein	Proteins containing a protein kinase family domain, c109925 and phosphotransferase family with PF 01636	
*B. bifidum* S17	YP_003938274.1^j^	BBIF_0495	Hypothetical protein	Proteins containing a protein kinase family domain, c109925 and phosphotransferase family with PF 01636	
	YP_003938776.1^k^	BBIF_0997	Mucin de-sulfatase	Proteins containing a protein kinase family domain, c109925 and phosphotransferase family with PF 01636	
	YP_003939526.1^l^	BBIF_1747	AG transferase	Phosphotransferase enzyme family of the PF 01636 and AG phosphotransferases of the aph family cd 05150	
*B. bifidum* PRL 2010	YP_003970614.1^j^	BBPR_0470	AG phosphotransferase	Proteins containing a protein kinase family domain, c109925 and phosphotransferase family with PF 01636	
*B. bifidum* BGN4	YP_006393921.1^j^	BBB_0447	AG phosphotransferase	Proteins containing a protein kinase family domain, c109925 and phosphotransferase family with PF 01636	
	YP_006394449.1^k^	BBB_0978	N-acetyl hexosamine kinase	Proteins containing a protein kinase family domain, c109925 and phosphotransferase family with PF 01636	
*B. bifidum* NCIMB 41171	ZP_07801902.1^j^	BBNG_00382	Conserved hypothetical protein	Proteins containing a protein kinase family domain, c109925 and phosphotransferase family with PF 01636	
*B. angulatum DSM* 20098	ZP_04447474.1^m^	BIFANG_02451	Hypothetical protein	Proteins containing a protein kinase family domain, c109925	
*B. animalis* subsp. *lactis* HN019	YP_002469703.1^e^	BLA_0835	AG phosphotransferase	Proteins containing a protein kinase family domain, c109925 and phosphotransferase family with PF 01636	
	ZP_02963731.1^e^	BIFLAC_04950	Hypothetical protein	Proteins containing a protein kinase family domain, c109925 and phosphotransferase family with PF 01636	
*B. animalis* subsp. *animalis* ATCC 25527	YP_006280402.1^e^	BANAN_06155	AG phosphotransferase	Proteins containing a protein kinase family domain, c109925 and phosphotransferase family with PF 01636	
	YP_006279244.1^n^	BANAN_00270	AG phosphotransferase	Phosphotransferase family with PF 01636 and aminoglycoside phosphotransferases of the aph family cd 05150	
*B. longum* subsp. *longum* JCM1217	YP_004221381.1^b^	BLLJ_1622	AG phosphotransferase	Proteins containing a protein kinase family domain, c109925 and phosphotransferase family with PF 01636	
*Bifidobacterium* sp. 12_1_47BFAA	ZP_07941182.1^b^	HMPREF0177_00575	Phosphotransferase enzyme family protein	Proteins containing a protein kinase family domain, c109925 and phosphotransferase family with PF 01636	
*B. longum* subsp. *infantis* 157F	YP_004209317.1^a^	BLIF_1400	Hypothetical protein	Proteins containing a protein kinase family domain, c109925	

^*^ Same superscript indicates proteins share >90% sequence percentage identity

AG: aminoglycoside

Proteins containing a protein kinase family domain, c109925, only and also annotated as aminoglycoside phosphotransferase or hypothetical proteins are also widely distributed across *Bifidobacterium* species. Some of these, such as BLD_0109 (*B. longum* DJ010A), Blon_0773 (*B. longum* subsp. *infantis* ATCC 15697) and BLJ_1379 (*B. longum* subsp. *longum* JDM301), are highly conserved while others, such as BLJ_1379 (*B. longum* subsp. *longum* JDM301) and BIFANG_02451 (*B. angulatum* DSM 20098), are more distantly related. Finally, 4 proteins (Bbr_0651, BIFBRE_03589, CECT7263_10981 and HMPREF9228_1217) were annotated as containing both a protein kinase family domain from c109925, while also containing a protein from the Pfam07462 (merozoite surface proteins). These 4 proteins were very highly conserved within the *B. breve* species sharing >99% percentage identity, while being more distantly related to proteins from other *Bifidobacterium* species, e.g. BIFANG_02451 from *B. angulatum* DSM 20098, which did not contain any protein of the Pfam07462.

We also investigated if the β-lactamases and aminoglycoside resistant protein sequences detected in bifidobacteria, could be classified according to the Ambler classes A-D for β-lactamases and acetylation, adenylation and phosphorylation enzymes for aminoglycosides. However, due to insufficient similarity with the sequences of known β-lactamases and aminoglycoside resistance proteins from other genera, such classifications were not possible. 

### Laboratory-based assessment of the antibiotic resistance of representative bifidobacterial strains

Laboratory tests were conducted with a number of representative *Bifidobacterium* species to determine if the presence of putative antibiotic resistance proteins corresponded to antibiotic resistance. The specific strains used had been determined, on the basis of the *in silico* screen, to contain putative β-lactam and/or aminoglycoside resistance genes. The use of different species and strains enabled us to determine if the results were genus, species or strain specific. The strains tested were *B. breve* UCC2003, *B. breve* DSM 20213, *B. gallicum* DSM 20093, *B. animalis* subsp. *lactis* Bb12, *B. angulatum* DSM 20098 and *B. pseudocatenulatum* DSM 20438 (Table 2). Disc diffusion assays were performed using both aminoglycoside [kanamycin (30µg), gentamycin (200 µg), streptomycin (25 µg) and neomycin (30 µg)] and β-lactam antibiotic discs [ampicillin (25 µg) and penicillin (10 µg)]. Following anaerobic incubation at 37°C for 48 hours, zones of inhibition were measured (Table 5). All tests were performed in triplicate. The results indicated that all strains tested were highly sensitive to the β-lactam antibiotics tested (all zones ≥ 52mm in diameter), thus establishing that the annotated β-lactamase genes did not confer resistance to the β-lactam antibiotics in the strains tested. Additionally, the β-lactamase nitrocefin tests also demonstrated a lack of β-lactamase activity among the bifidobacteria strains tested. In contrast, when these strains were tested using aminoglycoside antibiotic discs, each of the strains were shown to be highly resistant to each of the antibiotics, i.e. zone of inhibition was small or absent (Table 5).

**Table 5:** Antibiotic resistance of bifidobacteria strains as assessed through antibiotic disc assays.

**Antibiotic** (**microgram/per disc**)						
	**β-lactams**			**Aminoglycosides**		
**Bifidobacteria species**	**AMP 25**	**PEN 10 IU**	**KAN 30**	**GEN 200**	**STR 25**	**NEO 30**
*B. breve* DSM 20213	71mm	65mm	No zone	22mm	16mm	14mm
*B. animalis* subsp. *lactis* Bb12	65mm	55mm	No zone	28mm	21mm	20mm
*B. pseudocatenulatum* DSM 20438	61mm	56mm	8mm	10mm	13mm	20mm
*B. gallicum* DSM 20093	60mm	59mm	No zone	24mm	30mm	10mm
*B. angulatum* DSM 20098	64mm	65mm	4mm	23mm	16mm	10mm
*B. breve* UCC2003	67mm	56mm	No zone	26mm	21mm	10mm
*B. breve* UCC2003-0651-tet	52mm	57mm	10mm	40mm	33mm	14mm
*B. breve* UCC2003-1586-tet	62mm	57mm	9mm	41mm	31mm	15mm
*B. breve* UCC2003-1586-tet-pBC1.2-Bbr_1586	62mm	59mm	No zone	30mm	33mm	13mm

AMP, ampicillin; PEN, penicillin; KAN, kanamycin; GEN, gentamycin; STR, streptomycin; NEO, neomycin

Values are average of triplicate plate results (SD±1mm for all samples, on all antibiotics)

### Disruption of the *Bbr_0651* and *Bbr_1586* genes of *B. breve* UCC2003

An insertional inactivation approach was implemented to determine to what extent putative aminoglycoside resistance genes contribute to the observed aminoglycoside resistance in bifidobacteria. *B. breve* UCC2003 was selected as a target, due to the success with which gene disruptions have been previously created in this strain [50,51]. The genes *Bbr_0651* and *Bbr_1586* were targeted for disruption. The gene *Bbr_0651* encodes a putative conserved hypothetical secreted protein which shares 99% identity with other putative phosphotransferase enzymes (e.g. BIFBRE_03589 from *B. breve* DSM 20213) and also shares 71% identity with an aminoglycoside phosphotransferase from *B. longum* subsp. *longum* ATCC 55813 (HMPREF0175_1250). The gene *Bbr_1586* encodes a putative phosphotranferase family enzyme, which also shares 91% identity with a putative aminoglycoside phosphotransferase from *B. longum* *subsp.* *longum* ATCC 55813 (HMPREF0175_1250).

To determine if disruptions to the genes *Bbr_0651* and *Bbr_1586* which encode putative aminoglycoside resistance proteins impact on the aminoglycoside resistant phenotype of *B. breve* UCC2003, disc diffusion assays were carried out. Zones of inhibition were measured and compared to the wild-type, *B. breve* UCC2003. Differences in the inhibition zones were noted between the mutants and the wild-type, suggesting reduced aminoglycoside resistance in the mutants as compared to the wild-type *B. breve* UCC2003 (Table 5). Additionally, MICs were performed to compare aminoglycoside resistance of the wild-type to that of the two insertion mutants. As shown in Table 6, after 24 hours incubation, the insertion mutants were more sensitive to gentamycin, streptomycin and kanamycin, but not neomycin, as compared to the wild-type strain. These results thereby demonstrate that both *Bbr_0651* and *Bbr_1586* contribute to aminoglycoside resistance and can be assigned as aminoglycoside resistance determinants. To verify that the observed changes to phenotype were as a direct result of disruption to the genes *Bbr_0651* and *Bbr_1586*, rather than as an indirect consequence of the mutagenesis strategy, MICs were conducted on another insertion mutant created in *B. breve* UCC2003, namely *B. breve* UCC2003-*gos*G [51]. This mutant was created previously using the same protocol that was used to create the mutants *Bbr_0651* and *Bbr_1586*, but in this instance the *Bbr_0529* (*gosG*) gene is disrupted. The antibiotic resistance phenotype of this mutant was similar to that of the wild-type *B. breve* UCC2003 (Table 6). 

**Table 6:** MIC values (mg/L) of wild-type *B. breve* UCC2003 compared to mutants as determined by broth micro-dilution assay (MRS+cysteine for *Bifidobacterium* and LB broth for *E. coli* cultures).

	**Antibiotic (mg/L)**			
	**GEN**	**NEO**	**STR**	**KAN**
**Sample**	**1-1024**	**1-1024**	**2-4096**	**2-4096**
*B. breve* UCC2003 wild-type	>1024	>1024	1024	>4096
*B. breve* UCC2003-0651-tet	256	>1024	256	1024
*B. breve* UCC2003-1586-tet	256	>1024	256	1024
*B. breve* UCC2003-gosG	>1024	>1024	2048	>4096
*B. breve* UCC2003-1586-tet-pBC1.2-Bbr_1586	>1024	1024	256	4096
*B. breve* UCC2003 wild-type*	4096	4096	1024	4096
*B. breve* UCC2003-pBC1.2_Bbr_1586*	4096	4096	2048	8192
*B. breve* UCC2003-pBC1.2_Bbr_0651*	4096	4096	1024	4096
*B. breve* UCC2003-pBC1.2_Bbr_0651+0650*	4096	4096	1024	4096
*E. coli* XL1-blue-pBC1.2	<1	4	<2	<2
*E. coli* XL1-blue-pBC1.2_Bbr_0651+0650	2	8	<2	<2
*E. coli* XL1-blue-pBC1.2_Bbr_0651	2	8	<2	<2
*E. coli* XL1-blue-pBC1.2_Bbr_1586	<1	8	<2	<2

GEN, gentamycin; NEO, neomycin; STR, streptomycin; KAN, kanamycin

Values based on triplicate readings, which were identical in all cases

^*^ Higher ranges of antibiotics used to test effect of additional gene copies on MICs compared to wild-type (High range used: 256-16384mg/L for Gent/Neo; 1024-65536mg/L for Strep/Kan)

To further confirm that the observed reduction in aminoglycoside resistance of the insertion mutant was as a direct result of disruption to the putative AG resistance proteins, complementation studies were performed with one of the mutants. The MIC results demonstrate that following complementation, the resistance of the insertion mutant *Bbr_1586* was restored to levels almost identical to those of the wild-type (Table 6). Additionally, MICs were determined upon addition of extra plasmid-encoded copies of the putative aminoglycoside resistance genes *Bbr_0651*, *Bbr*_*0651+0650* or *Bbr_1586* into wild-type *B. breve* UCC2003 to determine if enhanced resistance to aminoglycosides would occur (Table 6). The results established that the addition of the construct pBC1.2-Bbr_1586 resulted in a 2-fold increased resistance to both streptomycin and kanamycin, relative to that of the parental strain. No increase in resistance to either gentamycin or neomycin was observed. Furthermore, the addition of either pBC1.2-Bbr_0651+0650 or pBC1.2-Bbr_0651 did not increase the resistance of UCC2003 to any of the tested aminoglycosides. Finally, the introduction of *Bbr_0651* or *Bbr_0651+0650* into *E. coli* XL1-blue resulted in a 2-fold increased resistance to gentamycin and neomycin, while the introduction of *Bbr_1586* also increased resistance to neomycin by 2-fold, relative to the control *E. coli* XL1-blue-pBC1.2 strain (Table 6). 

## Discussion

The human microbiota contributes to numerous vital gut functions including nutrient metabolism, vitamin biosynthesis and immune system development [52]. However, it has more recently been postulated that this complex microbial population is also a sizeable reservoir for antibiotic resistance genes [53,54], and that microbes containing such genes can become dominant in the human gastrointestinal tract following antibiotic exposure [36,55,56]. There is also a risk that such genes could be transferred to other microbes, including those passing through the gastrointestinal tract, and thus could contribute to the dissemination of antibiotic resistance genes [53]. Commensal bifidobacteria have received significant attention as a consequence of frequent reports of the beneficial impact of particular species or strains on health [25,57,58], with only one species, *B. dentium*, being a known human (cariogenic) pathogen [59]. Furthermore, given the frequent use of *Bifidobacterium* strains as probiotics, any association between these microbes and potentially transferrable antibiotic resistance would be a cause for concern. 

Several studies have utilised culture-based approaches to determine the resistance or sensitivity of bifidobacteria to various families of antibiotics, though the genetics underlying this resistance has not been examined extensively [29,31,35,43]. The exceptional studies that exist have focused on mutations to genes encoding specific targets and the resulting increased antibiotic resistance. In one instance the genetic basis for the enhanced resistance of mutants of *B. bifidum* Yakult strain YIT4007 was investigated [32]. Briefly, YIT 4007 was isolated from the progenitor strain YIT 4001 by screening mutants of YIT 4001 for enhanced resistance to neomycin, erythromycin and streptomycin. To investigate the potential transfer of resistance, genetic tests on the mutants were also performed. The study identified several chromosomal mutations, namely mutations on 3 copies of the 23S ribosomal RNA genes, an 8bp deletion of the *rluD* gene and a mutation on the *rspL* gene, which they considered to be responsible for the observed increased resistance to aminoglycoside antibiotics, at levels at which the progenitor strain was sensitive. As these mutations were not located on mobile genetic elements, it was concluded that this strain posed no risk of antibiotic resistance transfer. Another study investigated antibiotic resistance levels in 26 *B. breve* strains and found that a Yakult probiotic strain demonstrated atypically high resistance to streptomycin [37]. Genetic analysis determined that a mutation to the *rpsL* gene, which encodes the ribosomal protein S12, was responsible. In light of the general rarity of studies investigating the genetic basis for innate aminoglycoside resistance in bifidobacteria, this study examined the contribution of *in silico* assigned aminoglycoside resistance proteins to the resistance phenotype of bifidobacteria. Indeed, to our knowledge, ours is the first study that utilises a targeted *in silico* based approach to assess the existence and prevalence of putative β-lactamase and aminoglycoside resistance proteins in the *Bifidobacterium* genus and to subsequently investigate if representative genes confer a resistant phenotype. 

With respect to the putative β-lactamases, it was noted that several proteins of potential relevance have been assigned across the *Bifidobacterium* genus. However, none of these were clear representatives of any of the Ambler classes of β-lactamases. When all of the sequences were considered it appeared they could be grouped broadly into one of three groups, i.e. those which were members of Pfam 00144, those of Pfam 07521 or Pfam 12706. Most frequently these sequences were annotated as hypothetical proteins, while others were annotated as β-lactamases. To detect such a high prevalence of putative β-lactamases amongst bifidobacteria was surprising given that previous laboratory based investigations have shown bifidobacteria to be sensitive to commonly prescribed β-lactams [29,31,35,43,60]. Indeed, for example, in 2010 Xiao et al. demonstrated that 23 investigated bifidobacterial strains were sensitive to all β-lactams tested [31]. In order to examine whether these annotated β-lactamase sequences resulted in a resistance phenotype, we selected a representative number of bifidobacteria strains, which had been identified in the *in silico* screen as containing putative β-lactamases, and studied these further. Using a culture-based approach, the results indicated that none of the representative bifidobacterial strains which were tested were resistant to the β-lactam antibiotics. These results draw into question the significance of the high frequency of putative β-lactamases or hypothetical proteins closely related to β-lactamases in bifidobacteria genomes. The fact that the tested bifidobacteria were sensitive to β-lactam antibiotics and showed no β-lactamase activity (as assessed using the nitrocefin test), despite the presence of annotated β-lactams in their genome, as well as the lack of sequence homology when compared to known β-lactamase sequences, led us to conclude that this is most likely due to significant mis-annotation of protein sequences across publically available *Bifidobacterium* genomes. Alternatively, it could be proposed that these β-lactamase genes are repressed in bifidobacteria. While this possibility could be assessed by expression-based studies, which may be investigated in future studies, we think it more likely that the mis-annotation of these putative resistance genes is the basis for the absence of resistance. Indeed, there are previous examples of the mis-assignment of genes as penicillin resistance genes, such as the mis-annotation of the bile salt hydrolase genes as penicillin acylases [61,62]. With the development of high-throughput genome sequencing methods, automated approaches to annotation became increasingly popular [63]. However, this study provides an example of how mis-annotation of the first bifidobacteria genomes has led to further mis-annotation of subsequent genome sequences. Notably, several studies have investigated the extent of mis-annotation of genomes and noted the frequency of this issue [64-67], with one study finding an 8% error rate across just 340 genes [65]. Such an approach, which is likely to continue as sequencing becomes even more efficient and cost effective, and is coupled to automated annotation, could cause undue concern about the safety of a species, for example, in the case where antibiotic resistance protein sequences are detected in a potential probiotic bacterium. Thus, our results highlight the necessity for laboratory-based investigations into the function of annotated proteins.

Various culture-based studies have demonstrated that bifidobacteria are resistant to the aminoglycoside family of antibiotics [29,31,35]. This phenomenon was also apparent in the representative strains employed for this study. This resistance has been suggested to be due to the absence of appropriate cytochrome-mediated transport systems in bifidobacteria for aminoglycoside uptake [68]. This theory was first proposed in 1979, when it was demonstrated that *Bacteroides fragilis* and *Clostridium perfringens* were resistant to aminoglycoside antibiotics due to an inability to synthesize cytochrome structures and thus cannot utilise electron transport mediated transfer that is proposed to facilitate the entry of aminoglycosides into the cells [68]. It has since been accepted that bifidobacteria are intrinsically resistant to aminoglycoside antibiotics by the same mechanism [69]. However, we hypothesized that the resistance proteins detected in our *in silico* screen could be providing additional resistance beyond this intrinsic resistance and thus could contribute to the survival of bifidobacteria at higher concentrations of aminoglycosides.

The *in silico* screen highlighted the prevalence of putative aminoglycoside resistance proteins across members of the *Bifidobacterium* genus. Though a high frequency of aminoglycoside resistance proteins and related hypothetical proteins were detected, the sequences could be broadly categorised as those which were members of the Pfam 01636, those containing a protein kinase family domain c109925 and those which belonged to the Pfam 01636 and contained the domain c109925. To investigate the hypothesis that these putative resistance proteins contribute to aminoglycoside resistance in bifidobacteria, putative aminoglycoside resistance genes from one strain were mutated. More specifically, using *B. breve* UCC2003 as a representative strain, we disrupted the 2 genes present in this strain, which were detected in the *in silico* screen as being the genes potentially encoding aminoglycoside resistance proteins. Following confirmation that successful homologous recombination had occurred (at the targeted gene specific sites) within *B. breve* UCC2003, aminoglycoside resistance of the respective mutants was tested. These experiments demonstrated that disruption of either of these 2 aminoglycoside resistance genes impacted on the resistance phenotype of *B. breve* UCC2003 (Table 5). Thus, we propose that while the lack of cytochrome-mediated transport of the aminoglycosides into the cells may be an important contributor to the observed resistance phenotype among bifidobacteria and alone are sufficient to result in the strains being considered to be clinically resistant, these annotated aminoglycoside resistance proteins are true aminoglycoside resistance proteins, which further enhance this intrinsic resistance. To investigate this hypothesis further, MICs were conducted to compare the resistance of the mutants compared to the wild-type at higher levels of aminoglycoside antibiotics. The results established that the mutants exhibited greater sensitivities to gentamycin, streptomycin and kanamycin compared to the wild-type strain (Table 6). Unfortunately, the strategy employed precluded the creation of a double mutant that lacks both *Bbr_1586* and *Bbr_0651*. Should methods be developed to create deletion mutants in *Bifidobacterium* in the future, such a mutant can be created in order to determine if the inactivation of both aminoglycoside resistance genes results in a more pronounced aminoglycoside sensitive phenotype. Through complementation studies, it was demonstrated that reintroduction of the *Bbr_1586* gene restored resistance to gentamycin and kanamycin to levels which were essentially identical to those of the wild-type (Table 6). Additionally, when an extra, plasmid-borne copy of the gene *Bbr_1586* was added to wild-type *B. breve* UCC2003, a 2-fold increased resistance was seen for streptomycin and kanamycin. However, additional copies of *Bbr_1586* did not enhance resistance of the wild-type *B. breve* UCC2003 to neomycin and gentamycin. This may be due to the fact that the resistance of the wild-type to these antibiotics was already high (Table 6), and thus the aminoglycoside resistance proteins may have been saturated or unable to provide additional resistance to such high levels of antibiotics. Moreover, when an additional copy of either *Bbr_0651+0650* or *Bbr_0651* was added to the wild-type *B. breve* UCC2003, no additional enhanced resistance occurred for any of the aminoglycosides tested. This suggests that the genome-encoded copy of this gene is already performing its function optimally. The results in relation to *Bbr_1586* and streptomycin resistance are puzzling in that, while disruption to the putative aminoglycoside resistance genes resulted in a reduction in streptomycin resistance and additional plasmid-encoded copies of these genes increased the resistance to streptomycin compared to wild-type levels, complementation failed to restore streptomycin levels to those seen in the wild-type. One possible explanation is that there are additional genes downstream of *Bbr_1586*, which contribute to streptomycin resistance and are impacted upon in a polar manner following mutagenesis by plasmid insertion. The role of *Bbr_0651* and *Bbr_1586* as aminoglycoside resistance determinants was further confirmed through the provision of enhanced protection against at least one aminoglycoside upon their expression in *E. coli* XL1-blue. 

Ultimately, it is evident that both *Bbr_0651* and *Bbr_1586* contribute to aminoglycoside resistance in *B. breve* UCC2003. Importantly however, given that these resistance genes are not located on or near mobile genetic elements, they are unlikely to pose a risk of transferring antibiotic resistance to other bacteria populations. In fact it may be beneficial for species of *Bifidobacterium* to possess such non-transferable aminoglycoside resistance genes. Such species would survive higher levels of aminoglycosides than species without this additional genetic resistance, and so they may be more suitable as potential probiotics for use during aminoglycoside therapy. The results of this study re-emphasise the fact that annotation of genomes is a predictive process and that the results generated must be interpreted cautiously. Nonetheless, this approach did accurately predict the presence of aminoglycoside resistance proteins in bifidobacterial genomes. Crucially, laboratory based experiments were carried out to validate these annotations and similar such laboratory experiments are required to assess other putative antibiotic resistance genes in bifidobacteria and other genera.

## Supporting Information

Table S1 **Primers used in this study.**
(PDF)
