# Supplementary material for: In Silico Assigned Resistance Genes Confer Bifidobacterium with Partial Resistance to Aminoglycosides but Not to Β-Lactams
Source: PLoS One. 2013 Dec 6;8(12):e82653. doi: 10.1371/journal.pone.0082653 (PMC3855789; doi:10.1371/journal.pone.0082653)
Supplement: Table S1 — Primers used in this study. (PDF) [file pone.0082653.s001.pdf]

Table S1: Primer sequences used in this study

Primer name	Primer sequence	Source
**Primers for insertions mutants**		This study
Bbr_0651f (500bp insert product)	TGCGGAAAGCTTCGGTTCATCGCGAGGGACAG	This study
Bbr_0651r	CTATGCTCTAGACTCCATCTGCAACCATAG	This study
Bbr_1586f (400bp insert product)	TGCGGAAAGCTTGTATACAAATTCATCGAGCAC	This study
Bbr_1586r	CTATGCTCTAGAGCATGATGGTGTCGAGGTCG	This study
Pori19f	ATTGTGAGCGGATAACAATTTCAC	Law *et al.* (1995)
Pori19r	GATTAAGTTGGGTAACGCCAG	Law *et al.* (1995)
**Primers for complementation study and additional plasmid-encoded gene tests**		
Bbr_0651fhind3	TCGCTTAAGCTTGCTGCGCTTGTTCCATGACC	This study
Bbr_0651rxbaI	GAACTGTCTAGACTCGTTGGTGCCCGTCGCCG	This study
Bbr_0651+ 0650r2xbaI	CTGCCATCTAGAGACCGATGAGGCCACCGTG	This study
Bbr_1586fhind3	TCGCTTAAGCTTGAGACCTTCGACCTTCAGCCCAG	This study
Bbr_1586xbaI(1586 complement strain)	GAACTGTCTAGAGCGCCGCCCGTAACCAGAACAGT	This study

References

Law, J., G. Buist, A. Haandrikman, J. Kok, G. Venema, and K. Leenhouts. 1995. A system to generate chromosomal mutations in Lactococcus lactis which allows fast analysis of targeted genes. Journal of bacteriology **177:**7011-7018.
